# Synovial volume vs synovial measurements from dynamic contrast enhanced MRI as measures of response in osteoarthritis

**DOI:** 10.1016/j.joca.2016.03.015

**Published:** 2016-08

**Authors:** A.D. Gait, R. Hodgson, M.J. Parkes, C.E. Hutchinson, T.W. O'Neill, N. Maricar, E.J. Marjanovic, T.F. Cootes, D.T. Felson

**Affiliations:** † Wolfson Molecular Imaging Centre, The University of Manchester, Manchester, UK; ‡ Centre for Imaging Sciences, Institute of Population Health, The University of Manchester, Manchester, UK; § Arthritis Research UK Centre for Epidemiology, Centre for Musculoskeletal Research, Institute for Inflammation and Repair, Manchester Academic Health Science Centre, The University of Manchester, Manchester, UK; ‖ NIHR Manchester Musculoskeletal Biomedical Research Unit, Central Manchester University Hospitals NHS Foundation Trust, Manchester Academic Health Science Centre, Manchester, UK; ¶ Warwick Medical School, The University of Warwick, Coventry, UK; # Central Manchester University Hospitals NHS Foundation Trust, Manchester, UK; †† Department of Rheumatology, Salford Royal NHS Foundation Trust, Salford, UK; ‡‡ Clinical Epidemiology Unit, Boston University School of Medicine, Boston, MA, USA

**Keywords:** Osteoarthritis, Pain, Magnetic resonance imaging, Synovitis, DCE-MRI, Synovial volume

## Abstract

**Objective:**

Synovium is increasingly a target of osteoarthritis (OA) treatment, yet its optimal measurement is unclear. Using dynamic contrast enhanced (DCE) MRI in knee OA patients before and after intraarticular steroid injection, we compared the responsiveness of static synovial volume measures to measures of dynamic changes in synovial enhancement, changes that are strongly related to synovial vascularity.

**Methods:**

Ninety three patients underwent DCE-MRI before and 1–2 weeks after intra-articular injection of 80 mg methylprednisolone. Synovium was segmented and volume, relative enhancement rate (RER), maximum relative enhancement (RE_max_), late relative enhancement (RE_late_) and pharmacokinetic parameters (K^trans^, v_e_) were calculated. KOOS (​knee injury and osteoarthritis outcome score) pain score was recorded before and after injection. Standardized change scores were calculated for each parameter. Linear regression and Pearson's correlations were used to investigate the relationship between change in MRI parameters and change in pain.

**Results:**

The change in standardized score for the measures of synovial enhancement, RE_late_ and RE_max_ were −0.58 (95% CI −0.79 to −0.37) and −0.62 (95% CI −0.83 to −0.41) respectively, whereas the score for synovial volume was −0.30 (−0.52 to −0.09). Further, change in knee pain correlated more strongly with changes in enhancement (for both RE_max_ and RE_late, r_ = −0.27 (95% CI −0.45 to −0.07)) than with changes in synovial volume −0.15 (−0.35 to 0.05).

**Conclusion:**

This study suggests DCE-MRI derived measures of synovial enhancement may be more sensitive to the response to treatment and more strongly associated with changes in pain than synovial volume and may be better outcomes for assessment of structural effects of treatment in OA.

## Introduction

Synovitis is a common finding in the knees of patients with painful osteoarthritis (OA). Pain decreases after intra-articular steroid treatment and this may be related to effects on the synovium^1^. MRI is well suited to assessing synovial changes and by using intravenous contrast agent to differentiate synovial fluid from enhancing synovitis, the volume of enhancing synovitis can be measured.

In knee OA, the synovial volume has been found to be related to macroscopic thickening, proliferation and vascularization of the synovium^2^. Synovial biopsies show that volume is correlated with inflammatory cell sub-synovial lining infiltration, vascular congestion, proliferation of lining cells, fibrosis of synovial tissue and fibrin deposition on the synovial surface^3^. This would suggest that synovial volume in OA should shrink when the knee is treated with a potent anti-inflammatory such as intra-articular steroids and that change in synovial volume might be a good outcome measure for studies of anti-inflammatory treatments of OA. We recently demonstrated^4^ that synovial volume shrinks modestly after an intraarticular steroid injection but that the change in volume explained only around 3% of the reduction in knee pain experienced. Dynamic contrast enhanced (DCE) MRI, where images are acquired every few seconds after contrast injection, allows assessment of the time-course of contrast enhancement.

DCE-MRI has been used to study rheumatoid arthritis (RA)^5^, ^6^, where the rate and degree of enhancement of the synovium by contrast has been found to correlate more strongly with evidence of inflammation than the synovial volume^7^, ^8^, ^9^. DCE-MRI characteristics have been shown to be associated with RA in patients with early arthritis^10^. The histological features correlated with contrast enhancement include infiltration of inflammatory cells into the sub-synovial layer and especially vascular proliferation. DCE-MRI has also demonstrated a response to various different treatments in RA^11^, ^12^, ^13^, ^14^. It has also been used to investigate OA in the hand^15^ where it has shown a response to treatment in erosive OA of the interphalangeal joints^16^.

The aim of this study was to determine whether measures of synovial enhancement could detect a response to treatment and how this compared to changes in synovial volume. Structural features that are responsive to treatment in OA are being sought and we hoped that, like in RA, dynamic contrast enhancement might change more with treatment than synovial volume. An additional aim of our study was to investigate the relationship between MRI changes and changes in pain. If dynamic enhancement is more sensitive to change and better correlated with pain change than synovial volume, this would suggest that it is a better outcome measure for OA trials and that change in vascularity is critical to synovial response with anti-inflammatory treatments.

## Patients and methods

### Subjects

Men and women aged 40 years and over were recruited from both primary and secondary care clinics for participation in an open label study looking at the efficacy of intra-articular steroid therapy in symptomatic knee OA (ISRCTN: 07329370)^17^. Subjects were included if they reported moderate knee pain for more than 48 h in the previous 2 weeks. Inclusion criteria included imaging confirmation of significant OA radiologically defined as Kellgren and Lawrence grade 2 or greater in any compartment of the knee on anterior–posterior, skyline or lateral knee radiographs obtained within the previous 2 years. If X-rays were not available, persons could be eligible on the basis of findings on MRI scan or at arthroscopy. MRI and arthroscopy required typical changes of OA with at least cartilage loss not just fibrillation present. Exclusion criteria included the presence of secondary OA from gout, previous septic or inflammatory arthritis, injection with hyaluronic acid or steroid injection within the previous 3 months, history of knee surgery within the previous 6 months, concurrent life threatening illness, any contraindication to MRI scanning, absence of enhancing synovial tissue on MRI or estimated glomerular filtration rate (eGFR) less than 44 ml/min. Subjects were provided with an information sheet about the study and those who agreed to take part subsequently provided written informed consent. Ethics approval was received from the Leicestershire Multicentre Research Ethics Committee.

### Screening and baseline assessment

Knee radiography was performed in subjects who had not had a knee radiograph in the previous 2 years or other imaging evidence of OA. Those who were eligible were invited to attend for a baseline visit. Subjects completed also a series of questionnaires including the KOOS (​knee injury and osteoarthritis outcome score) pain scale at each time point (the primary symptom outcome). In addition, at each time point, we asked participants to identify the activity that caused the most knee pain and to score their current pain with this activity using a 0–10 visual analog scale (a secondary outcome).

### Intervention

Following the MRI scan, arthrocentesis was performed using an 18G needle carried out by one of two experienced clinicians (TON/NM) using a medial approach to the knee joint. Using the same needle the knee was then injected with 80 mg methylprednisolone (without local anesthetic). Synovial fluid obtained was forwarded for analysis and any subject in whom the synovial fluid white cell count was found to be greater than 1500/mm^3^ was subsequently withdrawn from the study because of concern that they may not have OA.

### MRI scan

Contrast enhanced MRI of the knee was performed just before intra-articular corticosteroid injection and then as close as possible to 10 days afterward as possible, a time frame corresponding to the time of maximal response to intraarticular steroids^18^. We studied one knee per person (the more symptomatic knee). Contrast agent administered intravenously using a power injector was Gadolinium-DOTA (Dotarem^®^, Guerbet, Paris, France) at a dose of 0.2 ml/kg.

Imaging was performed using a 3T Philips MRI scanner with a knee coil, unless the knee was too large for the knee coil when a FLEX-L coil was used instead. A sagittal pre-contrast 3-dimensional WATSc image was acquired (TR 20 ms, echo time (TE) 4.7 ms, field of view (FoV) 15 cm × 15 cm, 90 slices at a thickness of 1.5 mm, flip angle 15°, matrix size 272 × 272, pixel bandwidth 433 Hz). The standard dynamic dataset was acquired using a 3D fast field echo (FFE) sequence (TR 5.3 ms, TE ∼1.5 ms, FoV 14 cm × 14 cm, 20 slices at a thickness of 3 mm, flip angle 30°, matrix size 256 × 228, pixel bandwidth 543 Hz), (standard sequence). Eighteen 3D images were acquired at intervals of approximately 22 s. Intravenous contrast agent was administered between the third and fourth images. Patients who were too large for the knee coil were imaged using a modified 3D FFE dynamic sequence (SPGR: TR 9.3 ms, TE ∼1.5 ms, FoV 14 cm × 14 cm, 20 slices at a thickness of 3 mm, flip angle 30°, matrix size 256 × 228, pixel bandwidth 543 Hz) (lower temporal resolution sequence, LTR). Twelve images were acquired at intervals of approximately 39 s. Contrast agent was administered intravenously between the second and third images. A post-contrast T1 weighted fat suppressed turbo spin-echo image was acquired approximately 10 min after enhancement (TR 550 ms, TE 20 ms, FoV 14 cm × 14 cm, 24 slices at a thickness of 3 mm, ETL 3, matrix size 288 × 229, SPIR fat suppression, pixel bandwidth ∼240 Hz).

One hundred and twenty subjects had pre and post injection contrast enhanced images, data on synovial tissue volume (STV) were available for 111 at baseline and follow-up. In nine subjects no post-contrast sagittal image was taken or the quality of the image was considered to be poor, precluding comparison. Of these 111 patients, eleven knees were excluded due to incomplete or inadequate images, for example due to subject movement or failure to tolerate the examination, and a further seven did not show enhancing synovitis.

### Image analysis: segmentation

**Fig. 1:**
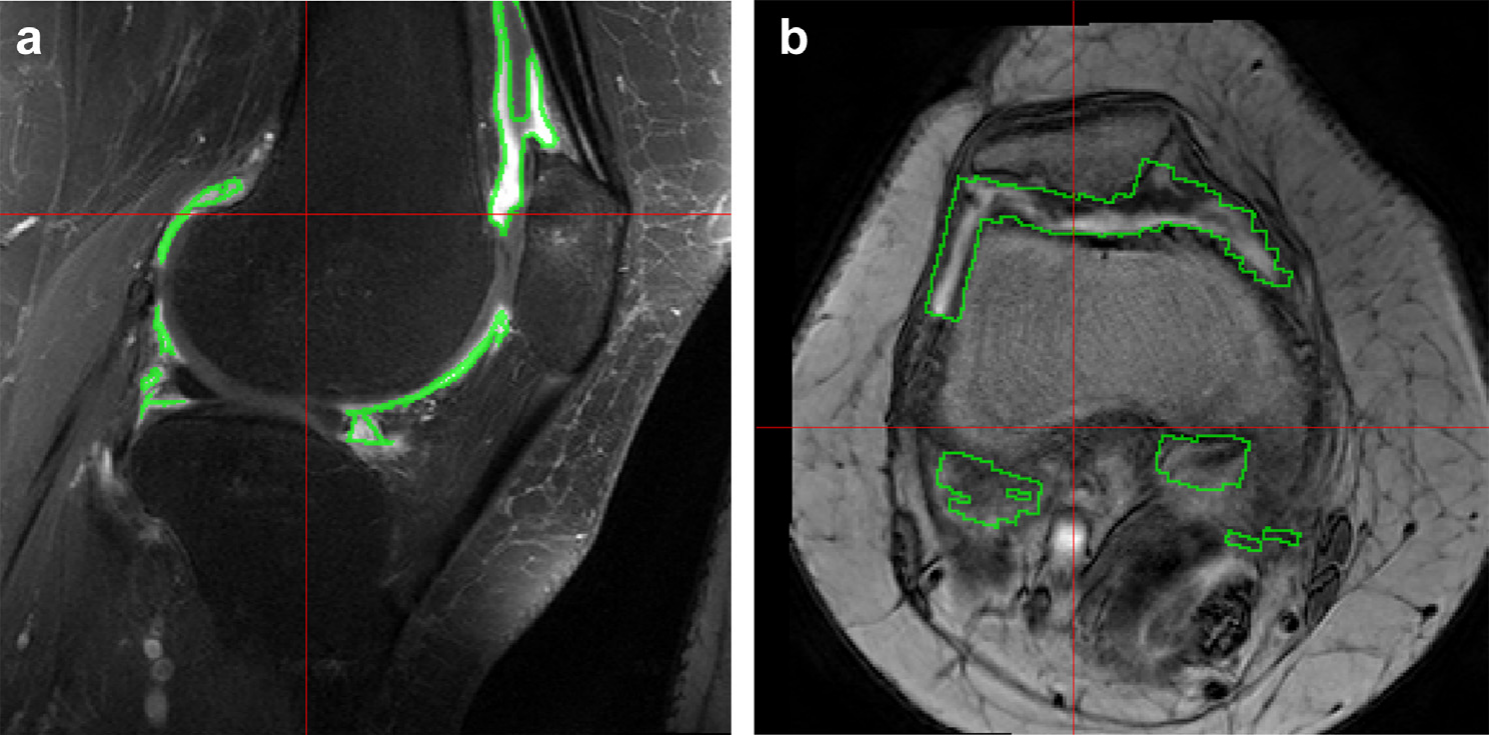
(a) Sagittal post contrast T1-weighted fat suppressed turbo spin-echo image after intravenous contrast enhancement with the synovial segmentation ROI outlined in green. Manual segmentation of the synovial tissue was performed on the sagittal post-contrast T1W FS image by a single observer. Segmentations were carried out paired, blinded to order. Using computer image analysis, cartilage was excluded by thresholding using the sagittal pre-contrast scan and the synovial fluid using the sagittal post-contrast scan (see also Ref. 4) The synovial volume is shown in Table II. (b) axial image from the DCE MRI sequence showing the placement of the ROI outlined in green. The ROI was determined from the pre and post contrast sagittal images and was then transferred to the axial dynamic dataset (truncated by the field-of-view of the dynamic images) for the DCE-MRI analysis.

Synovial volume was measured on the high resolution post-contrast images (see Fig. 1). Manual segmentation of the synovial tissue layer was performed on these sagittal post-contrast knee images by a single observer (interobserver ICC = 0.94), who assessed baseline and follow-up visit MR images paired, but blinded to order. Data on synovial volume in this study have been published including a description of our method of segmenting synovium to yield measures of synovial volume^4^.

### Image analysis: dynamic parameters

For each set of dynamic images, we calculated five parameters at each voxel of the image as follows:

Two parameters were calculated using the standard extended Tofts model^19^ with a population arterial input function (AIF) calculated by averaging values at each time point in the popliteal artery over all the patients in the study for whom this could be measured.

(a) K^trans^ (volume transfer coefficient)
(b) v_e_ (fractional extra-cellular extra-vascular space)

**Fig. 2:**
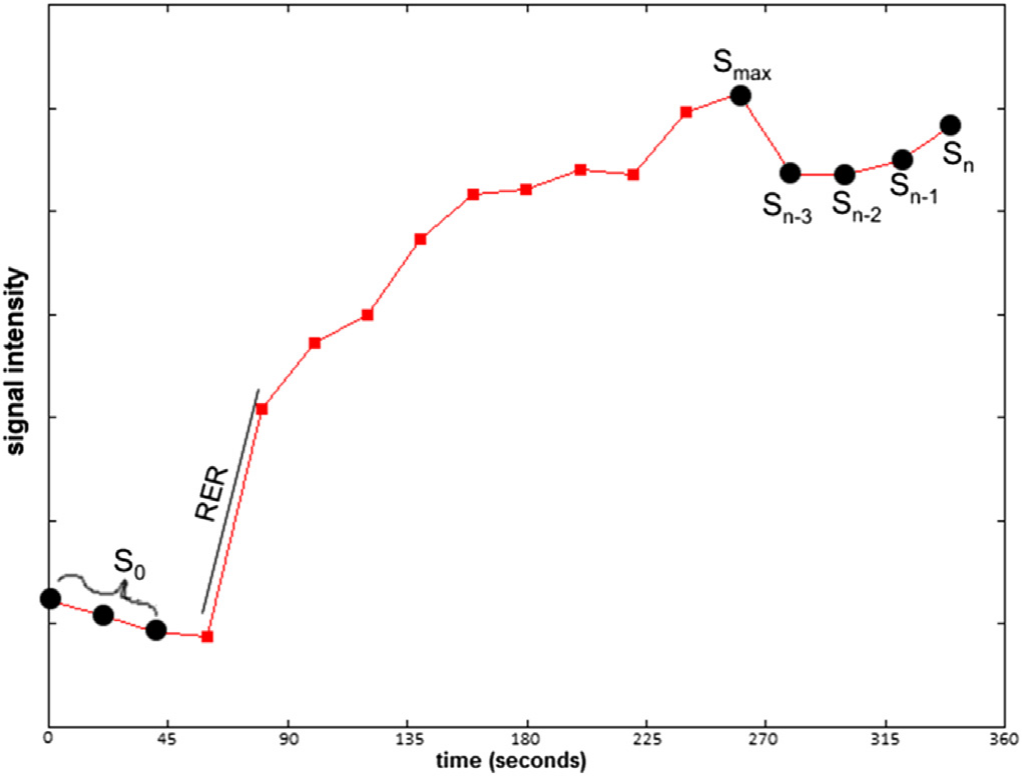
A typical time series S_i_(t) showing the values used to calculate parameters RER, RE_late_, and RE_max_.

Three parameters were calculated directly from the enhancement curve (see Fig. 2)

(c) the maximum relative enhancement rate (RER), which is defined as the maximum slope of the enhancement curve relative to the initial intensity.
(d) the maximum relative enhancement (RE_max_) defined as the maximum point of the enhancement curve relative to the initial intensity
(e) the late relative enhancement (RE_late_) = (S_n_+…+S_n−3_)/(4*S_0_) defined as the average of the last four points of the enhancement curve relative to the initial intensity

For each of these five parameter images, the manually segmented regions of synovium were overlayed onto the parameter images, via image registration, and the median value across all the voxels within these regions was calculated.

### Statistical analysis

The mean difference between the baseline and follow-up visit in MRI parameters plus the KOOS pain subscale, was calculated with 95% confidence intervals. This allowed assessment of the degree of change in each of the parameters at follow-up in the original units of measurement. To allow comparison of the magnitude of change across the parameters which used different scales of measurement, each parameter was firstly converted to a standard score (z-score). We then formed one ‘score’ variable with each parameter having a value at each time point. (K^trans^ to KOOS pain); each parameter was separated through the creation of additional dummy variables (collectively the ‘parameter type’). We then used random-effects multiple linear panel regression to assess the degree of change in each variable at follow-up, in terms of these z-scores. The model can be expressed formally as $y_{it}=X_{it1}\beta +X_{it2}\beta +W_{i}+\;U_{it}$, where *i* = participant ID as the panel (random-effects) variable, linking observations from the same participant, and *t* = the study visit (coded as either 0 for baseline, and 1 for follow-up visit) *y*_*it*_ = standard score variable, *X*_*it*1_ = parameter type (coded as a set of five dummy variables), *X*_*it*2_ = parameter type-by-study visit interaction effect (which allows for the standardized change to differ between the parameters), *W*_*i*_ = subject-specific random effect (which we assume is randomly distributed, and uncorrelated with the predictor variables), and *U*_*it*_ = error (which we also assume is randomly distributed). The unit of analysis for this model was therefore the patient. Using this method allows construction of 95% confidence intervals around the standardized change, unlike a more traditional standardized response mean.

Bivariate linear regression was additionally used to quantify the magnitude of relationships between the change in each of the MR measurements (dynamic parameters and synovial volume) and change in the KOOS pain subscale. For each of the six MR measurements, we carried out a bivariate linear regression model, with within-subject change in the KOOS pain subscale between baseline and follow-up as the outcome. The predictor variable in each model was the within-subject change in one of the six parameters at follow-up (e.g., change in RER). To examine the strength of correlation of pain change with change in MRI measures, we created Pearson's correlations (*r*) between change in the KOOS pain subscale and change in the variables of interest, so that we could compare across MRI measurements which were measured with different units. We also constructed 95% confidence intervals around these correlations. Statistical analysis was undertaken using Stata version 13.1.

## Results

We studied 93 patients (45.16% female, mean age 62.52 years, further characteristics in Table I). Every dynamic and static parameter showed a mean reduction in response to treatment between baseline and follow-up, although the mean percent reduction was less for synovial volume (15.4%) than for measures of enhancement such as maximal and late relative enhancement (24.4% and 24.6% respectively). The standardized score for change for the measures of synovial enhancement, RE_late_ and RE_max_ were −0.58 (95% CI −0.79 to −0.37) and −0.62 (95% CI −0.83 to −0.41), whereas for synovial volume it was −0.30 (95% CI −0.52 to −0.09) (see Table II).

Statistically significant associations were observed between pain change and change in the dynamic parameters RER, RE_late_, RE_max_ and K^trans^. However, the association between pain change and change in synovial volume was not statistically significant (see Table III). The correlations (*r* values) of pain change with change in synovium were greater for measures of late and maximal relative enhancement than for synovial volume.

## Discussion

The results of this study suggest changes in signal intensity measurements from synovial enhancement after intravenous contrast agent correlate with changes in pain after intra-articular steroid injection. The correlation was stronger than that observed between volume change and pain change. In the current study only the RER, maximum and late relative enhancement (RE_max_ and RE_late_) and K^trans^ change showed a significant association with pain change.

This study suggests that contrast enhanced intensity measures such as RE_max_ and RE_late_ may be more responsive than volume measurements for monitoring synovial change (Table II) and consequently may be better structural outcome measures for OA treatments targeting intraarticular inflammatory processes. Contrast enhancement depends on a number of factors such as synovial vascularity and the fractional volume of the extravascular, extracellular space into which the contrast diffuses which may change relatively acutely^19^. On the other hand, synovial volume may include regions of fibrous tissue which might be chronic.

We included subjects with grade 4 (severe) OA as recent work^20^ has suggested that they too show structural disease progression over time and that the synovitis and its severity are correlated with Kellgren and Lawrence disease grade^21^.

Although we found a stronger association of synovial signal intensity changes with pain than synovial volume with pain, this association was not strong. This compares with a previous 2.5 year observational study of 270 patients which showed a correlation of pain with synovitis change using unenhanced MRI of *r* = 0.2^1^. A previous cross-sectional study of 95 patients showed a significant correlation between DCE-MRI and pain (*r* = 0.4)^22^. There are likely to be many other factors apart from synovitis which influence pain such as bone marrow lesions, psychological issues, reduction in capsular stretching and others.

One reason why RE_max_ and RE_late_ correlated better than other measurements of contrast enhancement may be that they are relatively straightforward to measure and robust, in part because they do not require high temporal resolution. A previous study comparing OA and PsA in the hand also found late enhancement helpful^15^, although many studies have favored early enhancement rates^7^, ^8^, ^11^.

RE_max_ and RE_late_ behaved similarly in this study. That may reflect the enhancement curve characteristics (Fig. 2) which showed an increase then slow plateauing of signal intensity over the measurement period making RE_max_ and RE_late_ similar.

A measurement similar to RE_late_ could be obtained simply from a pre-contrast and a single delayed post-contrast image without requiring a rapid, dynamic imaging sequence. By reducing demands for temporal resolution, this would allow spatial resolution and coverage to be improved.

There have been a number of studies of treatment response in RA which have used measures of enhancement rate, maximum enhancement and/or delayed/static enhancement. The results have been mixed with the majority of studies demonstrating that measures of enhancement rate (this is similar to RER in the current study) and maximum enhancement^23^, ^24^, ^25^, ^26^ yielded similar trends. In most, stronger results were obtained from enhancement rates^23^, ^24^, ^25^, ^27^, ^28^, although in one, the maximum enhancement was more significant^29^. This may reflect the good correlation between enhancement rate and histology in RA^7^, ^8^, ^30^. The situation is further complicated by differences in definitions and temporal resolution. In OA, the situation may be different with lower rates of enhancement, particularly in the early enhancement phase^31^.

The use of intravenous contrast agent for accurately assessing synovitis is widely advocated, even though simple volume measurements are often made. The results of this study suggest that if intravenous contrast agent is being administered, signal intensity measurements may be more informative than synovial volume as they are more sensitive to change and correlate better with symptoms.

The study has a number of limitations. The low temporal resolution of the DCE-MRI sequence (22s and in some cases 39s) limits the accuracy of the model, particularly for estimating K^trans^. The field of view was also limited reflecting the compromise in DCE-MRI between temporal resolution, spatial resolution and field of view in the phase encode directions; these could be improved for measurement of late enhancement where high temporal resolution is less important. Also, a small subgroup of patients (16/93) was imaged using a different coil and different parameters due to large knee size. The differences in MRI repetition time (TR) are unlikely to have a significant effect on relative enhancement due to the strong T1 weighting of both sequences used. Furthermore, we have performed additional analyses which test for whether the magnitude of relationships differs between sequences, and found no significant differences between the sequence types (data not presented). Results from only those patients who had the standard imaging protocol (77/93) were similar. In addition, the joints were aspirated prior to injection. Aspiration may have resulted in apparent changes in measurements for both synovitis volume and enhancement due to alterations in partial volume effects. However, the reduction in both synovial volume and enhancement measurements in the time after aspiration and steroid injection suggests this was not the dominant effect. In addition, we assessed the correlation of pain change with synovitis change only in those with synovitis and by excluding seven patients without quantifiable synovitis, we may have overstated the association of pain change with synovitis change in all persons with knee OA. Further, in very large persons dynamic image sampling was less frequent and we could have missed a correlation of dynamic change with pain change.

Among possible other limitations include the multicollinearity of MRI parameters. Many of the dynamic parameters were strongly correlated (e.g., *r*_s_ = 0.98, between RE_max_ and RE_late_); therefore, while the results suggesting the superiority of dynamic vs static parameters are likely to be robust, our ability to distinguish the relative sensitivity of different dynamic parameters is limited. The time period between contrast injection and the acquisition of the post contrast static image used for segmentation limits the accuracy of the synovitis segmentation as contrast agent may have reached the synovial fluid^32^. The reproducibility of the different methods has not been assessed. Movement between dynamic images may degrade reproducibility of measurements such as RER. This could be reduced by use of image registration^33^. Other parameters such as the area under the enhancement curve were not calculated and these may also provide useful information.

While this study could be criticized as an open label and uncontrolled trial, our focus in this analysis was on comparing static vs dynamic measures and their relation to pain change, issues which should not be affected by the presence of a control group.

In conclusion, the results of this study suggest signal intensity based measurements obtained with contrast enhancement may be more appropriate outcome measures for synovitis in OA as they are more responsive and correlate better with symptoms than do measures of synovial volume.

Key messages:

1. Synovial measurements from DCE-MRI change more than synovial volume in response to intraarticular steroid treatment in OA.
2. Compared with synovial volume reduction, synovial measurements from DCE-MRI better correlated with pain relief.

## Data sharing and integrity

The corresponding author (DTF) had full access to all the data in the study, and takes responsibility for the integrity of the data, and the accuracy of the data analysis.

## Declarations of interest

Disclosures for DTF: Prof. Felson reported that he serves as a consultant for Zimmer Knee Creations.

## Contributions of authors

DTF initially proposed the study. Wrote the protocol: AG, RH, DTF, TFC. Wrote the manuscript: AG, DTF, MJP, RH. Collected the data: TWON, NM, RH, CEH, EJM, AG. Analysed the data: MJP, RH, AG. Reviewed drafts of the paper: CEH, TWON, MJP, NM, RH, AG, TFC, CEH, EJ.

## Competing interests

None.

## Financial support

NIHR Biomedical Research Unit, Arthritis Research UK and National Institutes of Health AR47785.

## Industry affiliations

None.

## Figures and Tables

**Table I:** Description of persons in study at baseline (unless otherwise stated)

Variable	Value	*N*
Age at baseline visit (years), mean (SD)	62.52 (10.69)	93
Females, frequency (%)	42 (45.16%)	93
Kellgren–Lawrence (K–L) Score	–	82†
- Grade 2, frequency (%)	29 (35.37)	
- Grade 3, frequency (%)	47 (57.32)	
- Grade 4, frequency (%)	6 (7.32)	
Number of days to follow up appointment, median (IQR)	8 (7–13)	93
Baseline KOOS pain subscale score∗ (0–100), mean (SD)	46.75 (14.40)	93

∗ Scoring for KOOS pain subscale is from 100 (no pain) to 0 (extreme pain).

† 11 patients were assessed for eligibility via MRI scan, rather than radiograph, and so had no K–L score.

**Table II:** Changes and standardized score for change of the parameters assessing the synovium

Variable name	Abbreviation	Baseline	Follow-up	Change at follow-up	Change in standardized score at follow-up
Mean (SD)	Mean (SD)	Mean (95% CI)	Mean (95% CI)
Synovial tissue volume (mm^3^)	V	9601 (5251)	8119 (4353)	−1482 (−2137 to −827)	−0.30 (−0.52 to −0.09)
Relative enhancement rate (min^−1^)	RER	0.048 (0.030)	0.032 (0.020)	−0.016 (−0.021 to −0.011)	−0.61 (−0.82 to −0.39)
Late relative enhancement	RE_late_	3.10 (1.37)	2.35 (1.12)	−0.76 (−0.98 to −0.54)	−0.58 (−0.79 to −0.37)
Maximal relative enhancement	RE_max_	3.51 (1.46)	2.65 (1.17)	−0.86 (−1.09 to −0.63)	−0.62 (−0.83 to −0.41)
Extravascular extracellular volume	v_e_	0.31 (0.22)	0.22 (0.23)	−0.09 (−0.13 to −0.05)	−0.39 (−0.60 to −0.17)
Volume transfer coefficient (min^−1^)	K^trans^	0.045 (0.033)	0.029 (0.028)	−0.016 (−0.022 to −0.010)	−0.51 (−0.72 to −0.30)
KOOS pain subscale score	–	46.75 (14.40)	69.68 (18.99)	22.93 (18.85–27.01)	−1.13 (−1.34 to −0.91)*

NB: *The KOOS pain score runs from 100 (no pain) to 0 (extreme pain).

**Table III:** Relation of change in pain with change in synovial parameters

Bivariate linear regression of change in [variable, below] with KOOS Pain subscale score∗		b coefficient (95% CI)	*P*	*r* (95% CI)
Synovial tissue volume (cm^3^)	V	−0.94 (−2.22 to 0.34)	0.15	−0.15 (−0.35 to 0.05)
Relative enhancement rate (min^−1^)	RER	−217.37 (−388.77 to −45.97)	0.01	−0.26 (−0.44 to −0.05)
Late relative enhancement	RE_late_	−5.08 (−8.83 to −1.32)	0.01	−0.27 (−0.45 to −0.07)
Maximal relative enhancement	RE_max_	−4.73 (−8.27 to −1.19)	0.01	−0.27 (−0.45 to −0.07)
Extravascular extracellular volume	v_e_	−9.96 (−29.89 to 9.98)	0.32	−0.10 (−0.30 to 0.10)
Volume transfer coefficient (min^−1^)	K^trans^	−151.18 (−299.73 to −2.63)	0.05	−0.21 (−0.39 to 0.00)

∗ Each of the variables in the table was entered into a bivariate regression against KOOS pain subscale change at follow-up visit, and subsequently, a bivariate Pearson's correlation. Note that the KOOS pain score runs from 100 (no pain) to 0 (extreme pain) so that a negative *r* value would signify that a reduction in synovial volume would be correlated with a reduction in pain. Note that all coefficients are negative. ‘b coefficient’ = unstandardized regression coefficient. ‘r’ = Pearson's correlation coefficient.

## References

[1] Hill C.L., Hunter D.J., Niu J., Clancy M., Guermazi A., Genant H. (2007). Synovitis detected on magnetic resonance imaging and its relation to pain and cartilage loss in knee osteoarthritis. Ann Rheum Dis.

[2] Loeuille D., Chary-Valckenaere I., Champigneulle J., Rat A.C., Toussaint F., Pinzano-Watrin A. (2005). Macroscopic and microscopic features of synovial membrane inflammation in the osteoarthritic knee: correlating magnetic resonance imaging findings with disease severity. Arthritis Rheum.

[3] Loeuille D., Sauliere N., Champigneulle J., Rat A.C., Blum A., Chary-Valckenaere I. (2011). Comparing non-enhanced and enhanced sequences in the assessment of effusion and synovitis in knee OA: associations with clinical, macroscopic and microscopic features. Osteoarthritis Cartilage.

[4] O'Neill T.W., Parkes M.J., Maricar N., Marjanovic E.J., Hodgson R., Gait A.D. (2016). Synovial tissue volume: a treatment target in knee osteoarthritis (OA). Ann Rheum Dis.

[5] Kubassova O., Boesen M., Boyle R.D., Cimmino M.A., Jensen K.E., Bliddal H. (2007). Fast and robust analysis of dynamic contrast enhanced MRI datasets. Med Image Comput Comput Assist Interv.

[6] Rastogi A., Kubassova O., Krasnosselskaia L.V., Lim A.K., Satchithananda K., Boesen M. (2013). Evaluating automated dynamic contrast enhanced wrist 3T MRI in healthy volunteers: one-year longitudinal observational study. Eur J Radiol.

[7] Ostergaard M., Stoltenberg M., Lovgreen-Nielsen P., Volck B., Sonne-Holm S., Lorenzen I. (1998). Quantification of synovistis by MRI: correlation between dynamic and static gadolinium-enhanced magnetic resonance imaging and microscopic and macroscopic signs of synovial inflammation. Magn Reson Imaging.

[8] Axelsen M.B., Stoltenberg M., Poggenborg R.P., Kubassova O., Boesen M., Bliddal H. (2012). Dynamic gadolinium-enhanced magnetic resonance imaging allows accurate assessment of the synovial inflammatory activity in rheumatoid arthritis knee joints: a comparison with synovial histology. Scand J Rheumatol.

[9] Vordenbaumen S., Schleich C., Logters T., Sewerin P., Bleck E., Pauly T. (2014). Dynamic contrast-enhanced magnetic resonance imaging of metacarpophalangeal joints reflects histological signs of synovitis in rheumatoid arthritis. Arthritis Res Ther.

[10] van de Sande M.G., van der Leij C., Lavini C., Wijbrandts C.A., Maas M., Tak P.P. (2012). Characteristics of synovial inflammation in early arthritis analysed by pixel-by-pixel time-intensity curve shape analysis. Rheumatology (Oxford).

[11] Meier R., Thuermel K., Noel P.B., Moog P., Sievert M., Ahari C. (2014). Synovitis in patients with early inflammatory arthritis monitored with quantitative analysis of dynamic contrast-enhanced optical imaging and MR imaging. Radiology.

[12] MacIsaac K.D., Baumgartner R., Kang J., Loboda A., Peterfy C., DiCarlo J. (2014). Pre-treatment whole blood gene expression is associated with 14-week response assessed by dynamic contrast enhanced magnetic resonance imaging in infliximab-treated rheumatoid arthritis patients. PLoS One.

[13] Boesen M., Kubassova O., Cimmino M.A., Ostergaard M., Taylor P., Danneskiold-Samsoe B. (2011). Dynamic contrast enhanced MRI can monitor the very early inflammatory treatment response upon intra-articular steroid injection in the knee joint: a case report with review of the literature. Arthritis.

[14] Cimmino M.A., Parodi M., Zampogna G., Boesen M., Kubassova O., Barbieri F. (2014). Dynamic contrast-enhanced, extremity-dedicated MRI identifies synovitis changes in the follow-up of rheumatoid arthritis patients treated with rituximab. Clin Exp Rheumatol.

[15] Schraml C., Schwenzer N.F., Martirosian P., Koetter I., Henes J.C., Geiger K. (2011). Assessment of synovitis in erosive osteoarthritis of the hand using DCE-MRI and comparison with that in its major mimic, the psoriatic arthritis. Acad Radiol.

[16] Jans L., De Coninck T., Wittoek R., Lambrecht V., Huysse W., Verbruggen G. (2013). 3 T DCE-MRI assessment of synovitis of the interphalangeal joints in patients with erosive osteoarthritis for treatment response monitoring. Skeletal Radiol.

[17] Arroll B., Goodyear-Smith F. (2004). Corticosteroid injections for osteoarthritis of the knee: meta-analysis. BMJ.

[18] O'Neill T.W., Parkes M.J., Maricar N., Marjanovic E.J., Hodgson R., Gait A.D. (2016). Synovial tissue volume: a treatment target in knee osteoarthritis (OA). Ann Rheum Dis.

[19] Tofts P.S., Brix G., Buckley D.L., Evelhoch J.L., Henderson E., Knopp M.V. (1999). Estimating kinetic parameters from dynamic contrast-enhanced T(1)-weighted MRI of a diffusable tracer: standardized quantities and symbols. J.Magn Reson Imaging.

[20] Guermazi A., Hayashi D., Roemer F., Felson D.T., Wang K., Lynch J. (2015). Severe radiographic knee osteoarthritis–does Kellgren and Lawrence grade 4 represent end stage disease?–the MOST study. Osteoarthritis Cartilage.

[21] Krasnokutsky S., Belitskaya-Levy I., Bencardino J., Samuels J., Attur M., Regatte R. (2011). Quantitative magnetic resonance imaging evidence of synovial proliferation is associated with radiographic severity of knee osteoarthritis. Arthritis Rheum.

[22] Ballegaard C., Riis R.G., Bliddal H., Christensen R., Henriksen M., Bartels E.M. (2014). Knee pain and inflammation in the infrapatellar fat pad estimated by conventional and dynamic contrast-enhanced magnetic resonance imaging in obese patients with osteoarthritis: a cross-sectional study. Osteoarthritis Cartilage.

[23] Reece R.J., Kraan M.C., Radjenovic A., Veale D.J., O'Connor P.J., Ridgway J.P. (2002). Comparative assessment of leflunomide and methotrexate for the treatment of rheumatoid arthritis, by dynamic enhanced magnetic resonance imaging. Arthritis Rheum.

[24] Axelsen M.B., Poggenborg R.P., Stoltenberg M., Kubassova O., Boesen M., Horslev-Petersen K. (2013). Reliability and responsiveness of dynamic contrast-enhanced magnetic resonance imaging in rheumatoid arthritis. Scand J Rheumatol.

[25] Tam L.S., Griffith J.F., Yu A.B., Li T.K., Li E.K. (2007). Rapid improvement in rheumatoid arthritis patients on combination of methotrexate and infliximab: clinical and magnetic resonance imaging evaluation. Clin Rheumatol.

[26] Axelsen M.B., Eshed I., Horslev-Petersen K., Stengaard-Pedersen K., Hetland M.L., Moller J. (2015). A treat-to-target strategy with methotrexate and intra-articular triamcinolone with or without adalimumab effectively reduces MRI synovitis, osteitis and tenosynovitis and halts structural damage progression in early rheumatoid arthritis: results from the OPERA randomised controlled trial. Ann Rheum Dis.

[27] Axelsen M.B., Ejbjerg B.J., Hetland M.L., Skjodt H., Majgaard O., Lauridsen U.B. (2014). Differentiation between early rheumatoid arthritis patients and healthy persons by conventional and dynamic contrast-enhanced magnetic resonance imaging. Scand J Rheumatol.

[28] Ostergaard M., Stoltenberg M., Henriksen O., Lorenzen I. (1996). Quantitative assessment of synovial inflammation by dynamic gadolinium-enhanced magnetic resonance imaging. A study of the effect of intra-articular methylprednisolone on the rate of early synovial enhancement. Br J Rheumatol.

[29] Buch M.H., Boyle D.L., Rosengren S., Saleem B., Reece R.J., Rhodes L.A. (2009). Mode of action of abatacept in rheumatoid arthritis patients having failed tumour necrosis factor blockade: a histological, gene expression and dynamic magnetic resonance imaging pilot study. Ann Rheum Dis.

[30] Gaffney K., Cookson J., Blades S., Coumbe A., Blake D. (1998). Quantitative assessment of the rheumatoid synovial microvascular bed by gadolinium-DTPA enhanced magnetic resonance imaging. Ann Rheum Dis.

[31] Kirkhus E., Bjornerud A., Thoen J., Johnston V., Dale K., Smith H.J. (2006). Contrast-enhanced dynamic magnetic resonance imaging of finger joints in osteoarthritis and rheumatoid arthritis: an analysis based on pharmacokinetic modeling. Acta Radiol.

[32] Ostergaard M., Klarlund M. (2001). Importance of timing of post-contrast MRI in rheumatoid arthritis: what happens during the first 60 minutes after IV gadolinium-DTPA?. Ann Rheum Dis.

[33] Hill A., Mehnert A., Crozier S., Leung C., Wilson S., McMahon K. (2006). Dynamic breast MRI: image registration and its impact on enhancement curve estimation. Conf Proc IEEE Eng Med Biol Soc.

