# Corrigendum to “Synovial volume vs synovial measurements from dynamic contrast enhanced MRI as measures of response in osteoarthritis” [Osteoarthritis Cartilage 24 (2016) 1392–1398]

**DOI:** 10.1016/j.joca.2017.05.001

**Published:** 2017-08

**Authors:** A.D. Gait, R. Hodgson, M.J. Parkes, C.E. Hutchinson, T.W. O'Neill, N. Maricar, E.J. Marjanovic, T.F. Cootes, D.T. Felson

**Affiliations:** † Wolfson Molecular Imaging Centre, The University of Manchester, Manchester, UK; ‡ Centre for Imaging Sciences, Institute of Population Health, The University of Manchester, Manchester, UK; § Arthritis Research UK Centre for Epidemiology, Centre for Musculoskeletal Research, Institute for Inflammation and Repair, Manchester Academic Health Science Centre, The University of Manchester, Manchester, UK; ‖ NIHR Manchester Musculoskeletal Biomedical Research Unit, Central Manchester University Hospitals NHS Foundation Trust, Manchester Academic Health Science Centre, Manchester, UK; ¶ Warwick Medical School, The University of Warwick, Coventry, UK; # Central Manchester University Hospitals NHS Foundation Trust, Manchester, UK; †† Department of Rheumatology, Salford Royal NHS Foundation Trust, Salford, UK; ‡‡ Clinical Epidemiology Unit, Boston University School of Medicine, Boston, MA, USA

We have been notified by the authors that there was an error in the second sentence of the paragraph headed ‘*Image analysis: segmentation*’ on p. 1394 of the above article. The term interobserver should have been intraobserver.

The correct sentence is as follows:

Manual segmentation of the synovial tissue layer was performed on these sagittal post-contrast knee images by a single observer (intraobserver ICC = 0.94), who assessed baseline and follow-up visit MR images paired, but blinded to order.

The authors would like to apologise for any inconvenience caused.

