# Supplementary material for: Synovial volume vs synovial measurements from dynamic contrast enhanced MRI as measures of response in osteoarthritis
Source: Osteoarthritis Cartilage. 2016 Aug;24(8):1392–8. doi: 10.1016/j.joca.2016.03.015 (PMC4967444; doi:10.1016/j.joca.2016.03.015)
Supplement: Supplementary file 1 [file mmc1.docx]

**MRI from study showing sagittal and axial image.**

**
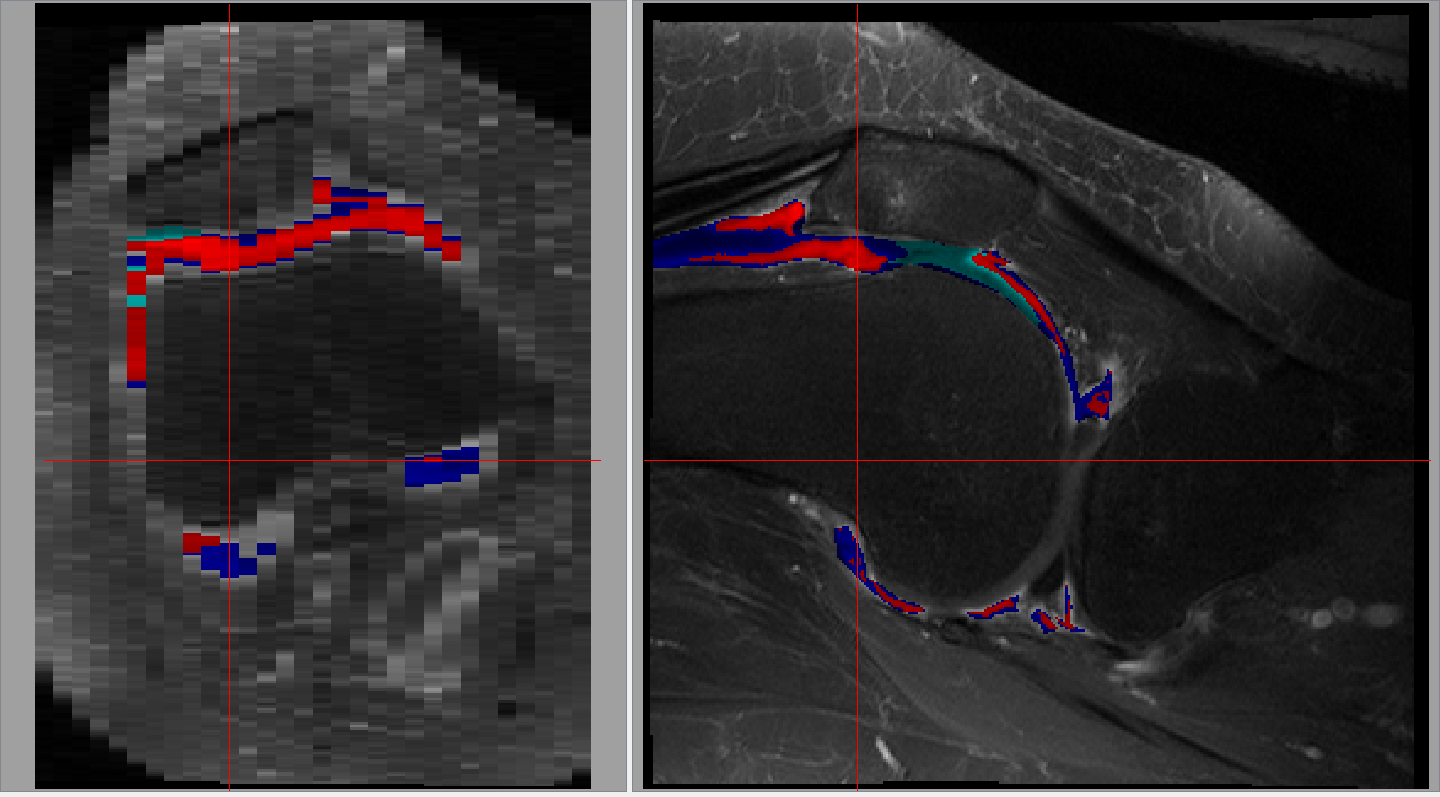
**

The axial was reconstructed from the sagittal coverage. Green indicates cartilage, red synovium and blue fluid.
